# High-Resolution Transcriptomic and Proteomic Profiling of Heterogeneity of Brain-Derived Microglia in Multiple Sclerosis

**DOI:** 10.3389/fnmol.2020.583811

**Published:** 2020-10-22

**Authors:** Anneke Miedema, Marion H. C. Wijering, Bart J. L. Eggen, Susanne M. Kooistra

**Affiliations:** Section Molecular Neurobiology, Department of Biomedical Sciences of Cells and Systems, University Medical Center Groningen, University of Groningen, Groningen, Netherlands

**Keywords:** multiple sclerosis, microglia, heterogeneity, spatial, sequencing

## Abstract

Microglia are important for central nervous system (CNS) homeostasis and first to respond to tissue damage and perturbations. Microglia are heterogeneous cells; in case of pathology, microglia adopt a range of phenotypes with altered functions. However, how these different microglia subtypes are implicated in CNS disease is largely unresolved. Multiple sclerosis (MS) is a chronic demyelinating disease of the CNS, characterized by inflammation and axonal degeneration, ultimately leading to neurological decline. One way microglia are implicated in MS is through stimulation of remyelination. They facilitate efficient remyelination by phagocytosis of myelin debris. In addition, microglia recruit oligodendrocyte precursor cells (OPCs) to demyelinated areas and stimulate remyelination. The development of high-resolution technologies to profile individual cells has greatly contributed to our understanding of microglia heterogeneity and function under normal and pathological conditions. Gene expression profiling technologies have evolved from whole tissue RNA sequencing toward single-cell or nucleus sequencing. Single microglia proteomic profiles are also increasingly generated, offering another layer of high-resolution data. Here, we will review recent studies that have employed these technologies in the context of MS and their respective advantages and disadvantages. Moreover, recent developments that allow for (single) cell profiling while retaining spatial information and tissue context will be discussed.

## Introduction

Multiple sclerosis (MS) is a common chronic neurodegenerative disease of the central nervous system (CNS) affecting 2.5 million people worldwide. MS symptoms include muscle weakness and spasms, movement difficulties, optic problems, fatigue, and acute or chronic pain (Koriem, 2016). In MS, demyelination contributes to axonal loss and gives rise to a neuroinflammatory environment. This provokes an immune response involving infiltrating immune cells and CNS-resident glial cells, including microglia, the innate immune cells of the CNS. Under homeostatic circumstances, microglia scan their surroundings with ramified processes and protect the nervous system from neural damage and inflammation. During pathology, microglia change their phenotype toward immune-reactive microglia. Morphologically, immune-reactive microglia acquire a more amoeboid morphology with retracted processes and secrete proinflammatory cytokines to aim for attenuated disease progression. A range of microglia phenotypes have been reported, possibly with distinct molecular and functional signatures, although these have not yet been fully elucidated.

In MS, demyelinated areas can be classified as preactive, active, chronic active, inactive, and remyelinated lesions, based on the degree of inflammation, demyelination, and axonal degeneration (Kuhlmann et al., 2017). This lesion heterogeneity is associated with alterations in microglia phenotype and functionality, giving rise to different microglia subtypes. However, the exact role and functions of different microglia subtypes in MS and other CNS diseases are largely unresolved. In addition, under healthy conditions, several factors contribute to microglial heterogeneity, for example the CNS region where the microglia reside in. Different subtypes of microglia are found in gray matter (GM) versus white matter (WM) (Anderson et al., 2007; McKay et al., 2007; van der Poel et al., 2019). Considerable variation in microglia subtypes and density has been described between brain regions as well. Lawson et al. (1990) reported variation in the density of microglia processes with a more than fivefold difference between brain regions. Furthermore, density variation was even observed within one brain region (Lawson et al., 1990), illustrating the importance of regional analysis. Why certain brain areas have a higher microglia density than others is not yet understood. In addition to microglia heterogeneity within an individual, microglia also differ between species and between male and female individuals, which until now mainly has been studied in mice and humans (Lenz et al., 2013; Torres-Platas et al., 2014; Galatro et al., 2017; Gosselin et al., 2017; Hanamsagar et al., 2017; Guneykaya et al., 2018; Villa et al., 2018; Geirsdottir et al., 2019). Furthermore, differences in the microglia transcriptome are observed when comparing microglia from various brain regions, and it has been shown that these regions are non-uniformly affected by aging (Grabert et al., 2016). Recently, high-resolution transcriptomic analysis, such as single-cell and single-nucleus sequencing, has contributed to a better understanding of microglia heterogeneity during development, in health, and disease (Keren-Shaul et al., 2017; Mathys et al., 2017; Brioschi et al., 2019; Hammond et al., 2019; Schirmer et al., 2019; Masuda et al., 2020).

Although these studies provided important insights and identified novel microglia subtypes, spatial information of the analyzed cells in their tissue of origin is lacking. In the past few years, technologies for the generation of gene expression that combine spatial information with gene expression data have been improved. Furthermore, single-cell proteomic methods, such as cellular indexing of transcriptomes and epitopes by sequencing (CITE-seq), are evolving. These methods will provide more insight into (regional) microglia heterogeneity under healthy circumstances and in case of CNS disease. Here, we will review recent studies in the context of MS that have implemented such methods and discuss their respective advantages and disadvantages.

## Microglia in MS

Multiple sclerosis is a heterogeneous disease in which different types of lesions are located in the CNS, with high variability in the number and type of lesions not only between patients but also within patients (Minneboo et al., 2005; Metz et al., 2014). Disease severity correlates with a higher lesion load (number of lesions per brain area), a higher number of chronic active lesions, and a higher proportion of foamy microglia/macrophages (Luchetti et al., 2018). Different cell types are involved in MS; a continuous cross-talk of astrocytes, oligodendrocytes, microglia, and neurons takes place within the brain. One of the key functions of oligodendrocytes is the production of myelin to ensheath axons for neurotrophic support and to facilitate optimal action potential transduction through the CNS (Kuhn et al., 2019). However, under pathological circumstances, these CNS cell–cell interactions are affected. Disturbances in the homeostatic environment initially lead to the activation of microglia, which is necessary for debris and pathogen clearance, repair, and formation of new cells to restore homeostasis (Peferoen et al., 2013). Also in MS, microglia get activated and migrate to the place of injury to clear debris, contribute to the repair of tissues/cells, and interact with surrounding neurons and astrocytes while secreting anti-inflammatory factors to dampen stress and promote remyelination (Luo et al., 2017; Prinz et al., 2019; Voet et al., 2019). However, activated microglia can also secrete proinflammatory factors that affect neighboring cells, including oligodendrocytes, resulting in oligodendrocyte death and production of poor-quality myelin sheaths (Luo et al., 2017; Masuda et al., 2020). In turn, oligodendrocytes produce chemokines, cytokines, and chaperokines, while astrocytes secrete proinflammatory genes, chemokines, and growth factors to control microglia activity, phagocytosis, and migration (Jha et al., 2019), illustrating a complex signaling network between cells during MS pathology.

### White Matter Microglia Lose Their Homeostatic Profile Upon Neuroinflammation Associated With MS Lesions

Neuroinflammation in the CNS is associated with a loss of the homeostatic microglia profile, which has been observed in various neuroinflammatory diseases (Dubbelaar et al., 2018). Beaino et al. (2017) have shown that, in mice, the local neuroinflammatory environment affects microglia activity by downregulating the expression of the homeostatic microglia marker *P2ry12.* Using human microglia *in vitro*, they identified that a proinflammatory environment decreases *P2RY12* expression (Beaino et al., 2017). This is supported by data that show a decrease in the level of P2RY12 in normal-appearing white matter (NAWM) and minimal P2RY12 immunoreactivity in active lesions in postmortem human MS tissue (Zrzavy et al., 2017). The same pattern of expression was seen for the homeostatic microglia marker *TMEM119* (Zrzavy et al., 2017; Van Wageningen et al., 2019). Interestingly, *P2RY12* reappeared in mixed active–inactive white matter lesions. This study revealed that messenger RNA (mRNA) levels of *P2RY12* and *TMEM119* are regulated by interleukin-4 (IL-4) and interferon-gamma (IFNγ). In contrast to white matter lesions (WMLs) and NAWM, levels of *P2RY12* and *TMEM119* did not differ between gray matter lesions (GMLs) and normal-appearing gray matter (NAGM). This could be explained by the lower number of lymphocytes observed within GMLs compared to WMLs, as lymphocytes secrete inflammatory mediators such as IL-4 and IFNγ and thus indirectly regulate *P2RY12* and *TMEM119* expression (Van Wageningen et al., 2019).

### Microglia in MS Lesion Pathology

**FIGURE 1:**
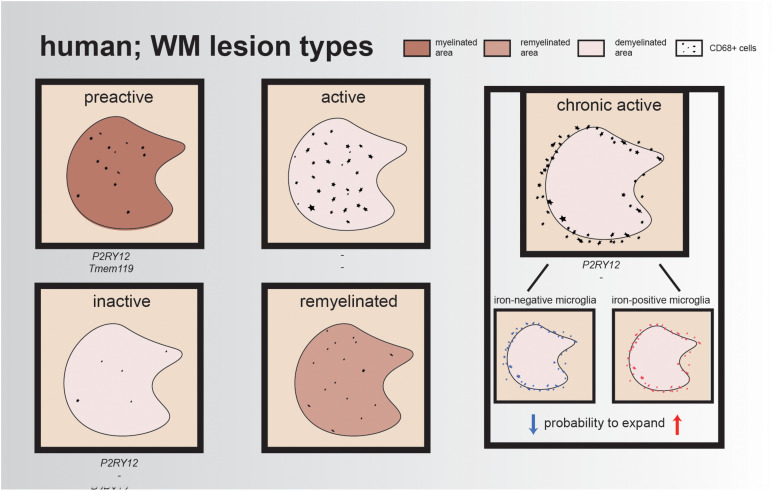
Illustrative overview of different human WM lesion types. Preactive lesions express the homeostatic microglia markers P2RY12 and TMEM119, while expression of these markers is minimal/absent in active lesions and reappears in chronic active lesions and inactive lesions; for remyelinated lesions, the expression of these genes remains unidentified. In each lesion type, CD68+ cells are represented, either within the lesion or at the rim of the lesion. The rim of chronic active lesions can either contain iron-positive microglia/macrophages resulting in a higher probability for lesion expansion or iron-negative microglia/macrophages, which often results in smaller lesions over time.

Lesions are often classified by the presence/absence of certain proteins to indicate de- or remyelination and/or inflammation. To classify these lesions, immunohistochemistry (IHC) can be performed using the inflammation markers human leukocyte antigen DR isotype (HLA-DR) and/or CD68 and a myelin marker, such as myelin proteolipid protein 1 (PLP1). Preactive lesions can be recognized by nodules of activated microglia (elevated levels of HLA-DR and CD68) in the absence of demyelination (van Horssen et al., 2012). These clusters of activated microglia express, e.g., tumor necrosis factor alpha (TNFα) and interleukin-10 (IL-10), which both play a role in cell survival, while IL-10 exerts also anti-inflammatory effects and is important for neurogenesis (Zhou et al., 2009; van Horssen et al., 2012; Pereira et al., 2015). Within these lesions, microglia have a ramified morphology and express the homeostatic markers P2RY12 and TMEM119, reflecting a (partly) homeostatic state (Figure 1).

In the healthy brain, oligodendrocytes and myelin are depositories of iron, an essential element for the regulation of myelination and oxidative phosphorylation (Hametner et al., 2013). However, in active MS lesions, oligodendrocytes are vulnerable to the inflammatory environment and, when damaged, release iron into the extracellular space, leading to the generation of reactive oxygen species (ROS) and uptake of iron by microglia and macrophages. Active lesions are characterized by a demyelinated core, containing an abundance of foamy myelin-containing microglia within the lesion. Another hallmark of active lesions is disruption of the blood–brain barrier (BBB), combined with leukocyte infiltration into the CNS (Kuhlmann et al., 2017; Grajchen et al., 2018). As a consequence, reactive microglia start synthesizing ROS, resulting in local oxidative stress, DNA damage, and neurotoxicity (Hametner et al., 2013, 2018; Yauger et al., 2019). These iron-laden microglia have the tendency to stay in this proinflammatory state, impairing clearance of myelin debris, making it harder for oligodendrocytes to migrate toward the lesion site and, as a result, complicating remyelination processes (Mairuae et al., 2011; Lee et al., 2019). Compared to active lesions, mixed active/inactive (chronic active) lesions contain significantly fewer infiltrated immune cells (Figure 1). Chronic active lesions can be recognized by a rim of HLA-DR-positive cells surrounding the demyelinated area. These reactive microglia contain phagocytosed iron and other phagocytosed products such as myelin and neuronal debris, which contribute to their amoeboid phenotype (Gillen et al., 2018). Dal-Bianco et al. (2017) visualized these lesions using magnetic resonance imaging (MRI) and showed that lesions with an iron-positive microglia/macrophage rim have a higher probability to expand than lesions with an iron-negative rim, which often become smaller, probably due to remyelination. This could indicate that, in lesions with iron-positive rims, remyelination is impaired (Dal-Bianco et al., 2017). Over time, the infiltrated immune cells disappear, resulting in a chronic silent lesion (inactive lesion). The majority of microglia in inactive lesions are positive for the homeostatic marker P2RY12, but these cells also still express proinflammatory factors (Figure 1) (Beaino et al., 2017; Zrzavy et al., 2017).

### Remyelination in MS

Cells from the oligodendrocyte lineage are important for remyelination within lesions. Remyelinated lesions show microglia polarization from a proinflammatory state toward an anti-inflammatory phenotype, necessary for the initiation of remyelination (Miron et al., 2013). First, a proinflammatory state is initiated to clear myelin debris and to stimulate oligodendrogenesis in newly formed lesions. When lesions expand, phagocytes acquire a more anti-inflammatory state and produce factors (IL-4, IL13, and IL-10) required for OPC differentiation (Butovsky et al., 2006; Miron et al., 2013; Cunha et al., 2020). This results in the de- and remyelinated regions that are often detected in/around active lesions. In remyelinated lesions, the so-called shadow plaques, iron accumulation is absent, suggesting that microglia present in these lesions have the capacity to clear myelin debris, enabling the maturation of OPCs, which results in the ability to remyelinate axons (Lampron et al., 2015; Dal-Bianco et al., 2017). In support of this notion, impeding myelin clearance through deletion of *Cx3cr1*, a receptor involved in microglia–neuron crosstalk, resulted in inefficient remyelination of axons (Lampron et al., 2015). Overall, microglia are very dynamic, and the environmental heterogeneity between and within MS lesions is associated with different microglia responses. Moreover, iron-positive microglia/macrophages that form a rim around the lesion could impair the remyelination process, resulting in slowly expanding lesions rather than shrinkage of the lesion.

## Heterogeneity of Microglia in the CNS

**FIGURE 2:**
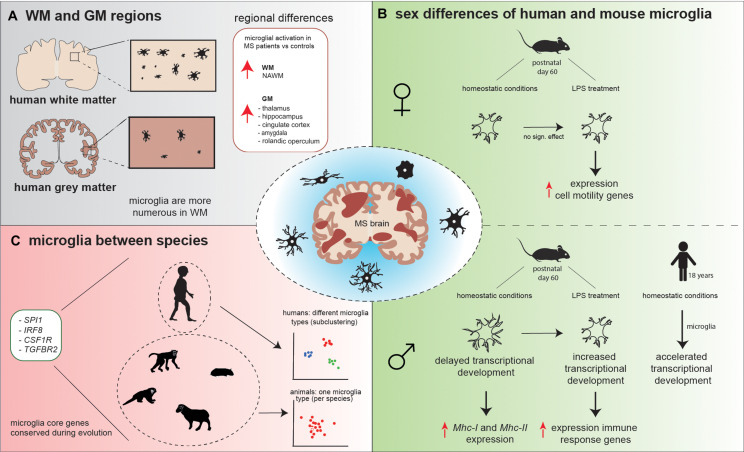
Schematic overview of microglial heterogeneity **(A)** in WM versus GM regions, **(B)** between human and mouse male and female microglia, and **(C)** between species. **(A)** Microglia in WM are more numerous compared to GM. Microglial activation is observed in NAWM, while in GM, microglial activation is conserved to specific areas. **(B)** Under homeostatic conditions, mouse male microglia have a higher process volume, process area, number of branches, and number of intersections compared to female microglia. These male microglia appeared to be delayed in the transcriptional development compared to female microglia and show increased expression of *Mhc-I* and *Mhc-II*. LPS treatment decreased male microglial total process volume and area, whereas in female individual, no significant effect of LPS on microglia morphology was observed. In response to LPS, male microglia increased their transcriptional development to a level that was comparable to the transcriptional development in female microglia observed prior to the LPS challenge and increased expression of immune response genes, while female microglia increased the expression of cell motility genes. In opposite, homeostatic human male microglia have an accelerated transcriptional development in comparison to female microglia. **(C)** *Spi1*, *Irf8*, *Csf1r*, and *Tgfbr2* are core microglial genes that are conserved during evolution, since these genes were strongly expressed in human, macaque, marmoset, sheep, mouse, and hamster. Human microglia subcluster into different microglial types, while mouse, macaque, marmoset, hamster, and sheep microglia grouped mainly into one microglia type, based on their gene expression profiles.

For a long time, research focused on WM pathology, as it was considered the main pathological characteristic of MS. However, demyelination and lesion formation is not restricted to the WM and also occurs in the GM (Petiet et al., 2016). In the last decades, studies expanded to include the GM due to advances in visualization technologies and IHC. Similar to WM lesions, cortical GM lesions can be subdivided into different lesion types: leukocortical (lesions that extend in subcortical WM), intracortical (lesions that do not extend to subcortical WM or the surface of the brain), and subpial lesions (lesions that extend to the surface of the brain). It has been shown that WM lesion load is related to cortical lesions; leukocortical and intracortical lesions correlated with the incidence of chronic active lesions, reactive site load, and the proportion of remyelinated lesions. However, no correlation was detected between WM lesion load and subpial lesions, indicating that subpial lesion formation is initiated by different underlying processes than in leukocortical and intracortical lesions (Luchetti et al., 2018). Interestingly, subpial lesion formation is often located close to sites of meningeal inflammation, which might contribute to GM lesion pathology (Howell et al., 2011). Moreover, differences in microglial phenotype, as well as transcriptomic differences, have been observed between species and male and female. Here, we focus on WM/GM microglial heterogeneity and microglial differences between species and sex (summarized in Figure 2).

### Microglia Are Heterogenous Across Brain Regions and Differ in WM Compared to GM Tissue

Microglia are present in both the WM and GM; however, these microglia are phenotypically different. Previous studies reported that microglia differ in density, morphology, and transcriptomic signature throughout the CNS, which could contribute to the difference in lesion pathology observed in WM versus GM (Lawson et al., 1990; Savchenko et al., 1997; Mittelbronn et al., 2001; de Haas et al., 2008; Böttcher et al., 2019; van der Poel et al., 2019; Van Wageningen et al., 2019). Furthermore, it has been observed that microglia show a region-specific gene expression profile; GM microglia were enriched for genes involved in type-I IFN responses, while WM microglia expressed high levels of genes involved in the nuclear factor kappa B (Nf-κb) pathway (van der Poel et al., 2019). Transcriptional differences were also observed in NAWM compared to NAGM; NAWM microglia increased the coexpression of genes involved in glycolysis and metal ion homeostasis (*ABCB6*, *CCR2*, *LPAR6*, *SDC1*, and *SLC25A37*), whereas in NAGM, microglia increased the coexpression of genes associated with lysosomal pathway, lipid catabolism, and foam cell differentiation (*ASAH1*, *CTSD*, *SCARB2*, *ABHD2*, and *LPL*).

Generally, microglia are more numerous in human WM than in GM (Figure 2) (Mittelbronn et al., 2001), which is in contrast to mice, where microglia numbers are higher in the GM (Kondo and Duncan, 2009; Lawson et al., 1990). However, microglia density is not homogeneous between specific WM and GM regions. In mice, the highest microglial density was detected in the frontal cortex, olfactory bulb, basal ganglia, and substantia nigra (regions low in myelin), while intermediate microglial density was detected in the cerebral cortex, thalamus, and hypothalamus, and microglial density was the lowest in the brainstem, cerebellum, and fiber tracts (myelin-rich regions) (Lawson et al., 1990). To determine whether microglia from WM or GM respond differently to the same lesion type, Cǎtǎlin et al. (2013) induced local tissue damage in both the WM (spine) and GM (cortex) with high-power laser pulses. The morphological response of WM and GM microglia did not differ upon this damaging cue. Although no difference in microglia morphology was identified, the speed at which microglia processes extended toward the lesion site (μm/min) was faster in WM compared to GM (Cǎtǎlin et al., 2013). Furthermore, Verdonk et al. (2016) used a Cx3cr1:GFP/++ model (mice in which the Cx3cr1 promoter drives expression of a GFP reporter gene) and treated one group with lipopolysaccharide (LPS). Twenty-four hours after LPS treatment, Verdonk et al. (2016) observed morphological differences in brain-derived microglia between the two groups; the cell body and cytoplasm area were significantly larger in microglia from the LPS group compared to controls. Interestingly, in both the LPS and control group, the cytoplasm area was significantly larger in the cerebellum compared to frontal cortex, hippocampus, and striatum. No significant differences were observed in cytoplasm area between these last three brain regions (Verdonk et al., 2016). Summarized, microglia are not uniformly distributed throughout the WM and GM brain regions. Microglia change their phenotype in response to immunomodulatory stimuli; however, differences are observed between brain regions.

#### Regional Differences in a Cuprizone Mouse Model

Cuprizone mouse models are frequently used to study de- and remyelination in WM and, to a lesser extent, in GM. Cuprizone is a toxic compound that induces oligodendrocyte death, resulting in robust demyelination. Mice on a cuprizone diet for a limited time are used as a model to resemble de- and remyelination in MS. Extensive demyelination occurs within the corpus callosum (CC); however, also GM areas such as the cortex are affected by demyelination (Skripuletz et al., 2008). A previous study from Gudi et al. (2009) showed that microglia start to accumulate in the CC 3 weeks after the start of the cuprizone diet and reach their peak at 4.5 weeks of treatment. In addition, a significant increase in the number of activated microglia was identified within the cortex; however, the increase was much lower compared to microglia accumulation within the CC (Gudi et al., 2009). Interestingly, heterogeneity in microglial distribution throughout the cortex was observed; at 3 weeks of cuprizone treatment, microglia mostly resided in cortical layer V, while at 4–4.5 weeks of treatment, microglia were equally distributed through both cortical layers V and VI. At weeks 5–5.5, the highest microglia number was observed in cortical layer VI, while at all these timepoints, demyelination was present in all cortical layers. Oligodendrocytes were also higher in number in these two layers of the cortex 4.5 weeks after the start of the cuprizone diet (Gudi et al., 2009). Cortical layers V and VI are most densely myelinated and are in proximity to the CC, which is one of the regions most affected by demyelination induced by cuprizone, possibly explaining the increase in microglia in these GM regions (Gudi et al., 2009; Tomassy et al., 2014). Another observation was increased microglial proliferation in both the CC and cortex during the demyelination phase. However, the number of proliferating cells decreased again during remyelination in both the WM and GM (Gudi et al., 2009). In MS lesions, remyelination is more extensive in the GM than in the WM, and the remyelination process occurs at a higher speed, which could be due to the higher number of OPCs present in GM lesions compared to WM lesions (Chang et al., 2012). Most cuprizone studies include young mice that receive a cuprizone diet for 5 weeks to investigate remyelination. In aged mice (6 months of age), 5 weeks cuprizone treatment did not result in complete demyelination; however, complete demyelination was achieved following 6.5 weeks of cuprizone treatment (0.4% cuprizone) with high accumulation of microglia within the CC. Interestingly, these mice showed incomplete remyelination, with increased numbers of microglia and prolonged microglial activation in the CC, even 3.5 weeks after withdrawal of cuprizone, which could suggest that aged microglia are less capable of phagocytosing debris, resulting in the delayed and incomplete remyelination seen in aged cuprizone mice (Gingele et al., 2020). Thus, when studying MS pathology, the use of an aged cuprizone mouse model should be considered, as this offers more reliable insights in de- and remyelination processes than the young cuprizone mouse model that is most commonly used.

### Microglia Differences Between Species

To study MS, cuprizone and EAE mouse models are commonly applied (Torkildsen et al., 2008; Constantinescu et al., 2011). However, animal models do not fully approximate or recapitulate human disease and pathology. First of all, when comparing mice to humans, we should take into account that the environment laboratory mice reside in is clean and controlled, while for humans, the environment changes frequently and contains microbes that could cause infections. Furthermore, genetic variation and environmental factors contribute to donor variation, while mice are generally inbred and most factors are held constant throughout an experiment. Here, we will discuss the differences in microglia phenotype and transcriptomic signature between species, with a focus on mice and humans.

Gosselin et al. (2017) compared mouse and human microglial transcriptomic data. Mice and humans showed an extensive overlap in microglial gene expression patterns (Pearson’s *r* = 0.806), although, genes involved in the regulation of the complement system and brain structure development were expressed at a higher level by human microglia (Gosselin et al., 2017).

Recently, a microglial gene expression profiling study of eight species across evolution—human, macaque, marmoset, sheep, mouse, hamster, chicken, and zebrafish—using a combined single-cell and bulk sequencing approach, was reported (Geirsdottir et al., 2019). Since chicken and zebrafish microglia showed evolutionary distance to the other six species, these two species were excluded from further analysis allowing the characterization of subtle microglial changes between mammalian species. Hierarchical clustering showed that the macaque transcriptomic signature was most similar to the human transcriptomic signature. Interestingly, all six species abundantly expressed *SPI1*, *IRF8*, *CSF1R*, and *TGFBR2*, genes with a critical role in microglial development, suggesting that these genes are core microglial genes that are conserved during evolution (Figure 2). In total, 163 genes were detected that were both conserved and specific for microglia. Moreover, it was observed that human microglia subcluster into different microglia types, based on their gene expression profiles, while mouse, macaque, marmoset, hamster, and sheep microglia grouped mainly into one type (Figure 2). One such human microglial subtype was identified by high expression of inflammatory genes, which could be a result of the effect of aging (Geirsdottir et al., 2019).

Our group previously reported that natural aging affects microglia differently in mice and humans. Just a small number of gene expression changes induced by aging are shared between mice and human microglia, and quite a few genes show opposite expression patterns (Galatro et al., 2017). Of note, although microglia age differently under healthy conditions, upon damage or disease, mouse and human microglia show very similar responses (Holtman et al., 2015b). The aging-related differences in expression patterns might be the result of external (signaling) factors, but also intrinsically, microglia could be dictated to express different genes upon aging. One of the processes possibly affecting microglia aging is their turnover rate. The microglia population is locally maintained by self-renewal. In adult rodents, it is unequal throughout the CNS and appeared to be higher in the dentate gyrus, where microglial proliferation declines faster with age compared to other brain regions. This could lead to an earlier-reached state of microglial senescence in this brain region, as a result of the Hayflick limit, which describes that cells have a finite capacity to divide due to telomere shortening; most cells divide around 40–60 times (Hayflick, 1965; Askew et al., 2017). This could explain the significant decrease in microglial proliferation rate that was observed in the dentate gyrus during aging. In humans, microglial proliferation rate has been estimated to be 2.9× higher compared to rodents (Askew et al., 2017). Furthermore, humans have a much longer lifespan than mice. When studying age-related brain diseases, it is important to take these differences into account, as they might influence the translational value from studies in mice to the human situation.

### Microglial Sex Differences

Women are at a two to three times higher risk to develop MS and suffer from more frequent relapses than men (Tremlett et al., 2008; Sellner et al., 2011). It was observed that MS characteristics are, among others, related to sex differences (Ribbons et al., 2015; Luchetti et al., 2018). In relapsing–remitting MS (RRMS), male individuals progress significantly faster than female individuals (Ribbons et al., 2015). In male individuals, a higher incidence of chronic active lesions was observed compared to female individuals. In addition, cortical lesions occurred more frequently in male than in female individuals. However, a direct relationship was observed between the presence of cortical lesions and disease severity in female individuals, while, interestingly, no such relation was observed in male individuals. Another observation was that the presence of chronic active lesions positively correlated with disease severity and lesion load (Luchetti et al., 2018), which could explain why in RRMS patients, a faster progression is observed in male compared to female patients.

So far, most research has focused on sex differences in T-cell immune responses in MS (Kodama and Gan, 2019). However, sex-specific differences have also been described for microglia. During embryonic and early postnatal development, mouse male and female microglia develop in a similar way based on gene transcription analysis (Hanamsagar et al., 2017). Microglia transcriptionally and morphologically reach a mature state at postnatal day 14, where they resemble homeostatic microglia from adult mice (Bennett et al., 2016; Matcovitch-Natan et al., 2016). However, in adult mice, it was observed that the increase in developmentally regulated genes during normal development is delayed in male microglia compared to female microglia, which can be used as an indicator for developmental maturation (Hanamsagar et al., 2017). When early adult mice were injected with LPS 2 h prior to isolation, changes in morphology, as well as at the transcriptional level, were observed in microglia. LPS treatment decreased male microglial total process volume and area, which was inversely correlated with gene expression changes normally associated with microglial developmental maturity. In female individuals, no significant effect of LPS on microglial total process volume and area was observed (Figure 2) (Hanamsagar et al., 2017). In addition, sex-specific gene expression responses were identified; male microglia adapted their expression of developmentally regulated genes to a level that was comparable to the expression of these genes in female microglia observed prior to the LPS challenge. LPS treatment increased the expression of immune response genes in male microglia, while in female microglia, cell motility genes were highly expressed (Figure 2). Female mice go through cyclic hormonal changes; however, different phases of the estrous cycle did not result in altered gene expression in microglia (Hanamsagar et al., 2017).

Interestingly, in humans, male microglia have an accelerated transcriptional development compared to female microglia (Figure 2) (Hanamsagar et al., 2017). Hanamsagar et al. (2017) hypothesized that this mouse–human difference could be a result of the relatively sterile environment that mice reside in. They postulated that males could be more susceptible to infectious agents during development than females, stimulating the transcriptomic development (Hanamsagar et al., 2017).

Morphological differences were observed when comparing microglia in early adult male and female mice (Lenz et al., 2013; Hanamsagar et al., 2017; Guneykaya et al., 2018; Villa et al., 2018). Differences in microglial process volume, process area, number of branches, and number of intersections were observed, which were all increased in males compared to females (Figure 2) (Hanamsagar et al., 2017). Guneykaya et al. (2018) also describe an increase in microglial density in the cortex, hippocampus, and amygdala in 13-week-old male mice. Furthermore, an increase in microglial soma size was seen in these three brain areas in adult male mice. These findings suggest that male microglia are in a more primed state and are prepared to directly react to immunomodulatory stimuli. This is supported by a higher expression of proteins involved in toll-like receptor (TLR) pathways found in male microglia, as determined by mass spectrometry-based proteomics (Guneykaya et al., 2018). In addition, *NF-κB* transcription activity was 2.4-fold higher in adult male microglia, also indicating a higher responsiveness to immunological stimuli (Villa et al., 2018). Thereby, adult male microglia have a higher antigen-presenting potential than adult female microglia. Using flow cytometry analysis, male microglia showed higher expression of *Mhc-I* in the hippocampus and amygdala, whereas *Mhc-II* expression was increased in the cortex, compared to the expression of *Mhc-I* and *Mhc-II* in female microglia (Guneykaya et al., 2018).

The different functional properties and the lower transcriptomic developmental state of human female microglia could be one of the factors contributing to the higher risk of MS in female individuals. In the last years, research groups have studied sex-specific features of microglia and their response to immunomodulatory stimuli. However, less is known about the effect of sex differences in microglia in disease and if these differences contribute to the development and progression of neurodegenerative diseases, such as MS.

### Analysis of Neuroinflammation in the Brain

In addition to the differences observed in microglial morphology in WM and GM, microglia change their phenotype during neuroinflammation. One method to analyze neuroinflammation is translocator protein (TSPO) positron emission tomography (PET) imaging. TSPO is present at the mitochondrial membrane, with increased expression in reactive astrocytes and proinflammatory microglia/macrophages (Chechneva and Deng, 2016; Beckers et al., 2018; Singhal et al., 2019). Under normal conditions, TSPO is expressed only at low levels in the GM in humans (Vowinckel et al., 1997; Banati, 2002). Using experimental-autoimmune encephalomyelitis (EAE), a mouse model for MS, Vowinckel et al. (1997) showed high concentrations of TSPO in the inflamed WM of EAE mice. In MS patients, significant differences in the uptake of a radioligand for TSPO were identified in specific regions of cortical GM areas compared to healthy controls; a recent study by Singhal et al. (2019) detected an increase in radioligand uptake in the hippocampus, amygdala, posterior cingulate, midcingulate, and rolandic operculum, indicating higher levels of microglial activation in these GM areas compared to healthy controls (Figure 2). However, microglia activation in total cortical GM did not differ from healthy controls (Singhal et al., 2019). A similar approach was used by Datta et al. (2017). Datta et al. (2017) detected that radioligand uptake predominantly occurred in the thalamus and that MS patients showed higher radioligand binding to TSPO in this region than healthy controls. Furthermore, an increase in radioligand uptake was identified in NAWM, compared to healthy WM (Datta et al., 2017), which is supported by Van Wageningen et al. (2019) who observed a decrease in the homeostatic gene expression of *P2RY12* and *TMEM119* in microglia, suggesting a shift toward reactive microglia.

All things considered, it is important to keep in mind that microglia are heterogenous in both WM and GM areas. Throughout the CNS, microglia show differences in density, morphology, and transcriptomic signature, with the latter being described more in detail in Chapter “Transcriptomic Profiling, an Initial Step Toward Understanding MS Heterogeneity.” In MS, higher levels of microglia activation can be found in a number of specific GM areas, while microglia activation levels are more or less homogeneous throughout the WM. Based on their gene expression profile, these human microglia subcluster into different microglial types, while in other species, microglia subcluster into only one microglial type. Moreover, male human microglia appeared to be more mature than female microglia, while interestingly, this is the other way around in mice. These male mice microglia have a more primed phenotype and a higher antigen-presenting potential than female mice microglia, and when responding to immunomodulatory stimuli, they push their transcriptomic signature to a more mature state. However, the question why some brain areas show higher microglial density and/or morphology compared to other brain areas still need to be answered.

## Transcriptomic Profiling, an Initial Step Toward Understanding MS Heterogeneity

### Low-Resolution Transcriptomic Profiling of Human MS Tissue

**FIGURE 3:**
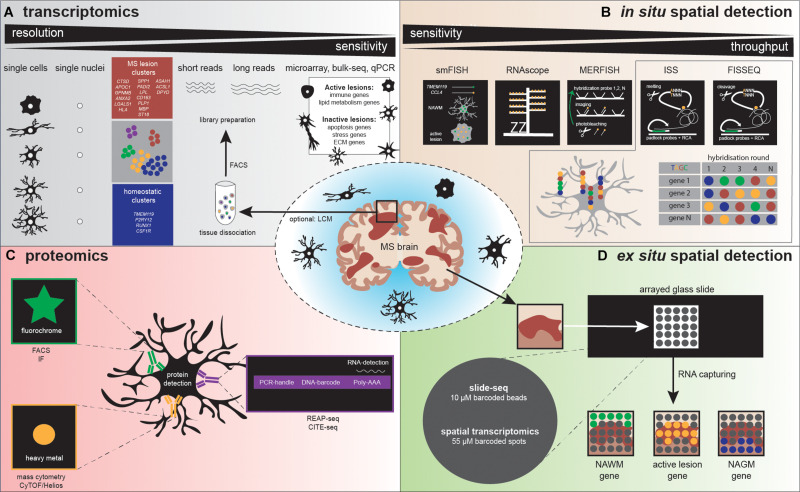
Illustration depicts various methodologies to detect microglia heterogeneity, **(A)** distinguished in transcriptomics, **(B)** *in situ* spatial detection, **(C)** proteomics, and **(D)** *ex situ* spatial detection. **(A)** After tissue dissociation and often FACS analysis, a library can be prepared to detect microglia heterogeneity. Sequencing detects heterogeneity at high (single cell/nucleus) resolution, but sensitivity is low, compared to microarray, bulk-seq, and qPCR. Several studies already detected homeostatic and MS-associated microglia clusters, characterized by the genes depicted in the blue and red squares, respectively. **(B)** The golden standard methods using a single probe (smFISH) and a probe with amplifiers (RNAscope) are followed by high-throughput *in situ* detection of genes, via multiple rounds of probe hybridization, cleavage, imaging, and finally sequence decoding. **(C)** Standardly applied proteomic methods use antibodies containing fluorophores to detect a protein, while high-throughput proteomics methodologies make use of antibodies labeled with heavy metals (mass cytometry) or DNA barcodes (REAP-seq/CITE-seq). REAP-seq and CITE-seq antibodies also contain an RNA binding site for simultaneous RNA detection. **(D)** A tissue section is placed on a barcoded slide with beads (slide-seq) or spots (spatial transcriptomics), which allows to explore regional gene expression in MS lesions.

Transcriptomic profiling of human MS postmortem brain tissues initially was performed using microarrays and bulk RNA sequencing approaches, which mainly resulted in information about neuronal cells, simply because neurons are more abundant than glial cells (Zheng et al., 2018). Therefore, combinations with laser capture microdissection (LCM), fluorescent-activated cell sorting (FACS), and region-specific analysis were applied to decrease sample heterogeneity. These studies identified gene signatures of active lesions (immune-related genes), which differ from the inactive lesion signature (apoptosis, stress-related genes) (Mycko et al., 2004; Elkjaer et al., 2019). An LCM-microarray study of chronic MS lesions detected increased expression of genes encoding for heat-shock proteins (HSP) in a region-dependent manner. Differences in specific *HSP* gene expression were observed between the margin and the center of the lesions, compared to NAWM. This lesion margin and center heterogeneity is potentially regulated by heat shock factor four (HSF4) (Mycko et al., 2012). In addition, inactive lesions increased expression of genes related to extracellular matrix components and steroid metabolism, while the rim of active lesions expressed genes related to lipid metabolism and lysosome signaling, including genes encoding for scavenger receptors (*OLR1*, *CD68*, *MSR1*, *CXCL16*), which are potential mediators of early demyelination (Figure 3) (Hendrickx et al., 2017). The role of lipid metabolism in early MS pathogenesis has been further elucidated, where lipid metabolism-related genes (*LPL*, *EEPD1*, *CHI3L1*) were enriched in NAWM compared to CWM (van der Poel et al., 2019). Besides lesion-specific profiles, a region-specific gene signature of the choroid plexus was identified, characterized by increased expression of hypoxia-related, neuroprotective, and secretory genes (Rodríguez-Lorenzo et al., 2020). Taken together, high molecular complexity is detected between lesion types, where the immune system and lipid metabolism are major processes linked with active lesions and apoptosis, while stress response and extracellular matrix changes are related to inactive lesions.

### High-Resolution Transcriptomic Profiling

Bulk transcriptomic profiling contributed to MS research, although its resolution by the analysis of pools of cells, masking cellular heterogeneity. Currently, a broad spectrum of sequencing technologies is available to analyze single cells (scRNAseq, see Figure 3 for an overview). Two types of amplification strategies can be distinguished: protocols that measure full-length complementary DNA (cDNA) molecules (SMART-seq2) (Picelli et al., 2014) and protocols that measure the 3′ or 5′ end of cDNA molecules linked to a unique molecular identifier (UMI) (MARS-seq, STRT, CEL-seq2, Dropseq, and inDrops). The use of UMIs massively increase the throughput; however, it decreases sensitivity, which remains higher in sequencing full-length cDNA. Combinations with microfluidics and droplet-based sequencing platforms (Fluidigm C1, Chromium-10X genomics and Indrop-Cellbio) created easy-to-use library preparation protocols. The advantages and disadvantages of these protocols are reviewed by See et al. (2018). For human CNS tissue, it is logistically challenging to obtain a viable single-cell population from fresh tissue prior to sequencing; therefore, some alternatives can be considered. If a sufficient number of cells can be obtained, possibilities exist to use a fixation buffer such as methanol, allowing temporal storage of single-cell suspensions at −80°. However, not all cells will recover from this process, and it should be tested if these buffers are suitable for glia cells (Chen et al., 2018; Böttcher et al., 2019). Instead of analyzing cells, individual nuclei isolated from fresh or frozen tissue can be profiled, a technique referred to as single nucleus RNA sequencing (snRNAseq) (Krishnaswami et al., 2016). The nuclear transcriptome of microglia has been reported to reflect the cellular transcriptome under homeostatic conditions and also following an LPS challenge (Gerrits et al., 2020).

#### Microglia Subtypes

Recently, high-resolution transcriptomic profiling (Figure 3) confirmed the existence of molecularly distinct microglia subtypes, with potentially specific functionalities (Keren-Shaul et al., 2017; Mathys et al., 2017; Geirsdottir et al., 2019; Hammond et al., 2019; Jordǎo et al., 2019; Masuda et al., 2019; Schirmer et al., 2019). To date, dynamics of microglial subtypes have been studied under homeostasis and during development, aging, and various neurodegenerative disease conditions in human and mouse models [including cuprizone and EAE, (de)(re)myelination and inflammation model for MS, respectively] (reviewed by Masuda et al., 2020). Complexity in microglial subtypes is increased in disease compared to homeostatic conditions and is highly influenced by the brain microenvironment. The availability of an extensive amount of transcriptomic profiles facilitated the separation of microglia from CNS-associated and blood-derived macrophages in gene expression datasets, based on marker genes (Jordǎo et al., 2019). scRNAseq/snRNAseq studies of microglia derived from human postmortem brain tissue from MS donors are scarce and only available with a limited number of donors and cells. However, CNS single-cell data are highly relevant for understanding MS pathogenesis. Masuda et al. were first to sequence individual CD45-positive cells isolated from MS brain tissue. Within their dataset, clustering analysis of 1,180 control microglia and 422 MS microglia identified 7 distinct clusters, all characterized by expression of *TMEM119* and *P2RY12*, referred to as microglia core genes. Controls mainly consisted of three clusters with high expression of microglia core genes linked to homeostatic functions. Homeostatic cluster four was present in controls and MS; this cluster decreased microglia core genes and increased cytokine and chemokine gene expression, referred to as preactivated microglia. In MS, three clusters were more abundant. The first cluster had increased expression of *CTSD*, *APOC1*, *GPNMB*, *ANXA2*, and *LGALS1*, while the second cluster was enriched for *HLA* genes related to antigen presentation, and the third cluster expressed genes that could be involved in demyelination: *SPP1*, *PADI2*, and *LPL* (Masuda et al., 2019). Recently, the first single-nuclei sequencing study in MS has been performed. In total, 1,524 MS microglia nuclei were compared to 159 control microglia nuclei. Hierarchical clustering distinguished microglia with homeostatic (*P2RY12*, *RUNX1*, *CSF1R*), MS-specific (activation markers, complement factors, *MHC-II* and lipid genes: *ASAH1*, *ACSL1*, *DPYD*), and phagocytosis- and oligodendrocytes-associated genes (*CD163*, *PLP1*, *MBP*, and *ST18*) (Schirmer et al., 2019). These studies indicate that unique gene expression changes occur in MS in specialized microglial subtypes. It is still unclear which factors initiate these gene expression changes and what is the exact functional role of these subtypes in MS. However, these studies provide us with candidate genes to be studied in more detail with functional assays, which likely contributes to future understanding of MS pathology. Taken together, high-resolution transcriptomic studies revealed microglia landscapes, showing the complexity of subtypes in a spatial and temporal manner. Extended information about transcriptional profiling of astrocytes, oligodendrocytes, and neurons in MS is available in Box 1.

**Box 1. MS-associated cell subtypes.**

Besides microglia, transcriptional profiling of other CNS cell types may contribute to understanding MS pathogenesis. Microglia interact with oligodendrocytes (Peferoen et al., 2013), astrocytes (Wheeler et al., 2020; Vainchtein and Molofsky, 2020), and neurons (Pósfai et al., 2019). In addition, in these cell types, transcriptional changes have been observed in human MS tissues. First of all, reactive *GFAP*-positive astrocytes are already transcriptionally distinct in MS NAWM tissue compared to controls. In MS NAWM, astrocytes are involved in responses to iron, oxidative stress, and immune responses, and they secrete factors for neuronal survival (Waller et al., 2016). Importantly, all above cell types are involved in (de)(re)myelination and immune signaling, which are the main processes that are altered in MS (Domingues et al., 2016; Molina-Gonzalez and Miron, 2019). In addition, recruitment of peripheral immune cells during MS pathogenesis is mediated by oligodendrocyte apoptosis and microglia immune activation (Chrzanowski et al., 2019). Therefore, high-resolution transcriptomic profiling of all cell types potentially involved in MS are relevant to understand MS pathology. Oligodendrocyte lineage cells are the actual suppliers of myelin, which was for a long term described as their only function as a pool of cells. However, high-resolution transcriptomics changed this passive view into a dynamic view, where oligodendrocyte lineage cells display heterogeneity across brain regions (Marques et al., 2016), throughout development (Perlman et al., 2020) and during the course of MS pathology (Jäkel et al., 2019; Schirmer et al., 2019; Yeung et al., 2019). As an example, Yeung et al. (2019) identified subsets of disease-specific oligodendrocyte lineage cells in EAE mice. One EAE-associated subtype was characterized by increased expression of *Mhc-II* and interferon-responsive genes, suggesting an active immune-modulatory role (Yeung et al., 2019). Functionally, they proved *in vitro* that Mhc-II-positive oligodendrocyte lineage cells performed phagocytosis (Yeung et al., 2019). Brain tissues from MS donors also contained MHC-II-positive oligodendrocyte lineage cells, and therefore, phagocytosis by these cells is likely involved in MS pathology. Others investigated human subcortical MS lesions, wherein a myelinating oligodendrocyte subtype increased the expression of genes associated with cellular stress (*HSP90AA1*, *FAIM2*, *ATF4*, and *UBB*), while myelin gene expression was reduced (*BCAS1*, *SGMS1*, *KCNJ10*, *SEMA6A*, and *GLDN*) (Schirmer et al., 2019). snRNAseq of various WM lesions derived from five MS donors generated a transcriptomic landscape consisting of six human oligodendrocytes subtypes. The frequency of each subtype differed within MS lesions and compared to control WM (Jäkel et al., 2019). In a similar approach that focused on astrocytes in EAE, astrocyte heterogeneity was detected and an EAE-associated astrocyte subtype, driven by increased *Mafg* and reduced *Nrf2* expression, was defined. This subtype was also present in 60% of the examined transcriptomic profiles of MS donors. In addition, immunohistochemistry showed that MAFG protein expression was increased in active human MS lesions, while NRF2 proteins already decreased in NAWM compared to controls. MAFG decreases the expression of NRF2, which is a negative regulator of inflammation and oxidative stress. Thus, when less NRF2 is available, inflammation is no longer inhibited. MAFG interacts with MAT2α to block anti-inflammatory pathways. Therefore, inflammatory responses increase due to this specific astrocyte subtype (Wheeler et al., 2020). In human tissue, isolation of astrocytes is technically challenging and, so far, has not resulted in the isolation of intact cells; therefore, analysis of astrocyte nuclei derived from frozen MS tissues is a major improvement. Nuclei sequencing resulted in a nuclear profile specific to subcortical WM (*SLC1A2*) and cortical GM (*CD44*) astrocytes, where all astrocyte populations expressed the marker *RFX4*. In the rim of active MS lesions, an increase in reactive astrocyte genes was observed (*BCL6*, *FOS*, *EDNRB*, *LINC01088*) (Schirmer et al., 2019). Schirmer et al. (2019) sequenced single nuclei of all CNS cell types, but their population consisted mainly of neurons. *CUX2* expressing upper-layer excitatory projection neurons (EN-L2-3A/B) showed major gene expression differences between MS and control donors and trajectory analysis defined a link with chronic inactive lesions. Functionally, *CUX2*-positive neurons were annotated with gene ontology (GO) terms related to oxidative stress, mitochondrial dysfunction, and cell death. Therefore, *CUX2*-positive neurons are sensitive to cell damage, and especially, this neuronal subtype is reduced in MS compared to other, preserved neuronal subtypes (Schirmer et al., 2019).

## Spatial Detection Could Provide New Insights in Regional Heterogeneity

High-resolution transcriptomics technologies have made it possible to study the entire transcriptome at the level of an individual cell. These methods are highly relevant for understanding MS pathogenesis, as they can detect differentially expressed genes in the affected tissue and potentially identify novel cell subtypes. However, these technologies require disruption of the tissue for the isolation of cells or nuclei from the tissue, and as a consequence, spatial context is lost. However, spatial context is important in understanding cell functioning in health and during pathology. Thus, techniques that allow spatial detection could provide added value when studying (regional) heterogeneity to further elucidate pathogenic features (Figure 3).

### *In situ* Hybridization Assays

In 1969, *in situ* hybridization (ISH) was pioneered by Pardue et al., and this was followed by the invention of fluorescent probes around 10 years later (Pardue and Gall, 1969; Rudkin and Stollar, 1977). (Fluorescence) *in situ* hybridization [(F)ISH] is a technique that uses labeled DNA or RNA probes to target a specific DNA or RNA sequence within a histological section, thus indicating gene expression at a spatial level. A problem that arose with this technique was that single molecules could only be detected in regions with a low background signal. In 1998, single-molecule FISH (smFISH) was developed by Femino et al. (1998). smFISH includes probes labeled with five fluorochromes that target multiple regions of the transcript, resulting in a higher fluorescence signal, allowing specific detection of single molecules (Femino et al., 1998). However, the use of probes consisting of five different fluorochromes was associated with a high risk of both false-positive and false-negative outcomes. Furthermore, these heavily labeled probes were difficult to develop (Femino et al., 1998; Asp et al., 2020). Raj et al. (2008) optimized smFISH. The technique uses 40 probes, each coupled to a single fluorochrome, resulting in more accurate mRNA counts (Raj et al., 2008). Hammond et al. (2019) studied microglial RNA expression patterns in mice during development, in old age, and after brain injury. Following demyelination injury by lysolecithin (LPC) injection, this research group used smFISH to determine the organization of microglia within these demyelinated lesions. Three probes were used for smFISH to label the microglial marker *Fcrls* and chemokines *Ccl4* and *Cxcl10*. smFISH confirmed an increase in Fc receptor-like molecules+ (Fcrls+) microglia and showed higher *Apoe* expression after LPC injection compared to control mice. Furthermore, smFISH identified a spatial distribution of *Ccl4* and *Cxcl10* within the lesion; both chemokines were expressed only in small specific parts of the demyelinated area, and in most of the cases, the chemokine expression colocalized with *Fcrls*+ microglia. In human MS active lesions, also an increase in *CCL4* expression was observed, mostly in TMEM119+ microglia (Figure 3) (Hammond et al., 2019). Another study investigated regional GM heterogeneity by studying neuronal gene expression in the different layers of the cortex, to further elucidate MS pathology (Schirmer et al., 2019). Using smFISH, probes for simultaneous detection of two to three genes of interest were included, and gene expression was measured in cortical GM, adjacent subcortical WM lesion areas, and controls. smFISH showed a decrease in upper-layer neuronal marker *CUX2* in completely and incompletely demyelinated cortical MS lesions, while expression of the interneuronal marker *VIP* was maintained. Furthermore, smFISH confirmed the increase in the neuronal cell stress markers *PPIA* and *NORAD*, which were mainly upregulated in demyelinated cortical MS lesions (Schirmer et al., 2019). A similar approach to smFISH is RNAscope. RNAscope uses target-specific double Z probes that target the mRNA. Then, amplifiers and different labels (chromogenic or fluorescent) can be added to these Z probes. Targeted mRNA transcripts are detected as dots, each dot representing a single copy of the mRNA. Compared to smFISH, RNAscope probes amplify signals while simultaneously suppressing the background noise (Wang et al., 2012).

In (sm)FISH, the number of fluorescent probes is generally limited to three due to limitations of fluorescent microscopy, such as the use of band- and long-pass filters (Lukumbuzya et al., 2019). Multiplexed error-robust FISH (MERFISH), a technique that was developed in 2015 allows for the detection of multiple probes. MERFISH uses multiple rounds of hybridization and imaging, with a different probe for each RNA molecule (Figure 3) (Chen et al., 2015). These probes label the RNA with a specific combination of readout sequences, resulting in “barcodes” assigned to the RNAs of interest. The barcodes are then read out by the use of fluorescent probes that target the RNA sequences. The higher the number of rounds, the higher the copy number of genes that can be measured simultaneously. To adjust for detection errors that exponentially increase with the number of rounds, error-robust encoding schemes were added that detect and correct errors to improve accuracy and sensitivity (Chen et al., 2015; Strell et al., 2019). A recent study investigated the role of astrocyte heterogeneity and regulation in MS (Wheeler et al., 2020). This group identified an astrocyte population characterized by an increase in MAFG expression and a decrease in NRF2 expression in EAE and MS. In addition, MERFISH revealed that these *Mafg*+ astrocytes were in close proximity to granulocyte-macrophage colony-stimulating factor *Gm-csf*+ T cells (Wheeler et al., 2020). Thus, MERFISH adds value when spatially investigating the (co-) expression of a high number of RNAs of interest simultaneously.

### Spatial *in situ* Sequencing Techniques

Fluorescent *in situ* sequencing (FISSEQ) is a method that was first described by Mitra et al. (2003). First of all, a library with molecules of linear DNA is generated. Each of these molecules contain one variable region that is linked to two constant regions. These constant regions allow primer binding during an amplification step. In-gel amplification of this library results in so-called polonies (polymerase colonies). During FISSEQ, the polonies are denatured, and sequencing primers hybridize to the template (Figure 3). Next, sequencing is performed by different cycles in which single fluorescent nucleotides are added and imaged (Mitra et al., 2003). In 2014, the next-generation of FISSEQ was developed (Lee et al., 2014). This technique allows the use of both fresh-frozen (FF) tissue and formalin-fixed paraffin-embedded (FFPE) tissue. First, the fixed tissue is tagged with random hexamers, followed by reverse transcription and cDNA amplification. Next, these cDNA amplicons are cross-linked *in situ* and sequenced. Each base is visualized in one color, resulting in certain barcodes that can be mapped to the genome. There is no need to select candidate genes as this method captures all of the mRNA and thus can localize the RNA whole transcriptome. Other advantages of this technology are high sensitivity and high throughput. Furthermore, FISSEQ can be applied to intact cells and tissues, avoiding sectioning. However, low-abundant targets are not detected by FISSEQ, as the mRNA to cDNA conversion efficiency is limited (Lee et al., 2014, 2015).

*In situ* sequencing (ISS) was first described in 2013 (Ke et al., 2013). The ISS procedure starts with reverse transcription of mRNA to cDNA, followed by the hybridization of padlock probes to the cDNA strand (Figure 3). Two approaches were developed: gap-targeted sequencing and barcode-targeted sequencing. Both approaches are followed by rolling-circle amplification and sequencing by ligation, which makes it possible to sequence small RNA fragments, within cells, and tissue sections. For gap-targeted sequencing, one extra DNA polymerase step has to be taken to fill the gap. The barcode-targeted sequencing approach uses a padlock probe containing a barcode. During sequencing by ligation, anchor primers are hybridized next to the targeted sequence. Then, sequencing probes containing a fluorescent label for one specific nucleotide bind to the anchor primers, allowing the decoding of the gap sequence or the barcode (Ke et al., 2013). In 2017, CARTANA commercialized ISS (barcode-targeted sequencing approach) promising the simultaneous detection of 600 target genes. To increase the detection sensitivity for low-abundant targets, multiple probes could be assigned to one target, as was performed by Chen et al. for the microglial gene *Itgam* (Chen et al., 2020). In this study, ISS was used as a validation method for spatial transcriptomics (ST), discussed below. ISS was performed in a mouse model for Alzheimer’s disease to determine if certain gene expression patterns could be localized to specific cell types. They observed that microglia were the main cell type responsible for expressing plaque-induced genes. Eighteen of these plaque-induced genes overlapped with disease-associated microglia (DAM) genes, which are also increased in MS mouse models (Krasemann et al., 2017). ISS thus could bring us new insights in MS pathology at a spatial single-cell level. Moreover, low-abundancy problems could be solved using multiple probes for one target.

### Spatial *ex situ* Sequencing Techniques

Another approach to profile gene expression while retaining spatial tissue information is spatial transcriptomics. ST was first reported by Ståhl et al. (2016) and was improved by 10X Genomics in 2019 under the name “10X Visium” with an increase in resolution. ST and 10X Visium make use of special glass slides containing barcoded mRNA-capturing probes printed in spots (Figure 3). All probes within one spot have the same spatial barcode. First, samples are fixed on the glass slide followed by hematoxylin and eosin (HE) staining and imaging. Next, the tissue is permeabilized, which allows RNA binding to the barcoded mRNA-capturing probes. This barcode will be incorporated into the cDNA during reverse transcription. The last step is library preparation and sequencing (Salmén et al., 2018). ST and 10X Visium thus combine histology and transcriptomics. An advantage of these techniques is that prior knowledge of certain genes is not needed, as the 200 million probes within a spot capture all of the mRNA in a tissue section. However, the resolution does not yet approach the single-cell level; the spots have a diameter of 100 μm (ST) or 55 μm (10X Visium), and thus, multiple cells (depending on the tissue type) are captured within each spot. The use of ST could be of interest when analyzing different lesion types in MS. Several studies already analyzed gene expression profiles of different MS lesions, for example by bulk-RNA sequencing or single-cell sequencing (Mycko et al., 2004; Elkjaer et al., 2019; Schirmer et al., 2019). ST/10X Visium retains spatial information by staining and imaging the tissue prior to tissue permeabilization. The spatial barcode makes it possible to map gene expression patterns back to their original location within the tissue section (Figure 3). ST/10X Visium thus could add insights in spatial gene expression profiles within the lesions itself or where the lesion transitions to NAWM. A limitation of ST technology is that it is not suitable for detection of low abundant targets, which is true for the majority of microglial genes. Furthermore, only a relatively small tissue section can be analyzed, as the mRNA-capturing probes lay in squares of around 6 × 6 mm (ST) or 8 × 8 mm (10X Visium) (Salmén et al., 2018).

A similar approach to ST/10X Visium is slide-seq, which was developed in 2019. Slide-seq also uses glass slides containing barcoded beads (Figure 3). These barcodes refer to a position on the glass slide, which makes it possible to map gene expression back to the imaged tissue section. However, in contrast to ST/10X Visium, adjacent tissue sections are used for staining and imaging, which could decrease the specificity when including heterogeneous tissues. A major advantage of slide-seq is that the barcoded beads have a resolution of 10 μm and can detect gene expression with approximate single-cell resolution (Rodriques et al., 2019; Asp et al., 2020). Furthermore, it takes about 3 h to process the tissue, while this is around 8 h for 10X Visium (for both techniques, this excludes imaging and quality control time) (Rodriques et al., 2019). However, slide-seq is not yet commercially available.

In summary, in the last few decades, different techniques have been developed that make use of spatial gene expression analysis. Methods that allow for spatial detection could provide information about heterogeneity of cells and genes within tissue sections, interactions between cells, and their function and cellular composition. These findings could contribute to novel insights in the underlying mechanisms of pathologies, which are crucial in the process to identify novel therapeutic targets. Depending on the study and its aim, it is important to keep in mind the advantages and disadvantages of the spatial technologies as sensitivity, resolution, and throughput differ between the spatial assays and could lead to wrong interpretation of research outcomes.

## Preanalytical Factors to Consider During Microglia Transcriptomics Profiling

Both high-resolution transcriptomics and spatial detection methods measure RNA levels. In addition, high-resolution transcriptomics requires isolation of microglial cells. It remains largely unclear how preanalytical factors such as RNA quality, postmortem delay (PMD), and microglia isolation methods exactly influence the microglial transcriptome. These preanalytical factors vary between labs, and standard procedures are lacking. In order to obtain a pure microglia cell population, mechanical or enzymatic tissue dissociation methods are required, followed by FACS or magnetic bead sorting. Alternatively, in mice, microglia-specific RNA molecules can be captured using RiboTag or PAPERCLIP technologies (Hwang et al., 2017; Haimon et al., 2018). Analysis of microglia gene expression by RiboTag identified that changes can occur upon microglia isolation, where proinflammatory genes are significantly upregulated due to dissociation at 37°C (Haimon et al., 2018). The PAPERCLIP technology has the advantage that microglia cells represent a more native state because alternative polyadenylation does not affect microglial gene expression profiles (Hwang et al., 2017). Furthermore, microglia can be obtained via LCM-guided single-cell isolation. However, the microglia transcriptome appeared to be different when comparing LCM- and FACS-isolated microglia, where LCM-isolated microglia are not a pure microglial population, since 50% neuronal and oligodendroglial transcripts were also detected (Solga et al., 2015). In mice, tissue dissociation steps can be skipped using the genetic cTag-PAPERCLIP or Ribotag model for microglia isolation, which more accurately reflects the microglia transcriptome (Hwang et al., 2017). Of note, spatial detection methodologies are always independent of microglia isolation procedures, therefore reflecting *in vivo* microglia more accurately; of course, the effect of PMD still remains. The Netherlands Brain Bank isolated microglia cells of 100 donors, including MS donors who, in general, have a longer PMD compared to controls, since an extended autopsy protocol was applied for MS donors using MRI to select lesions. RNA isolation and quantitative PCR (qPCR) were performed on microglial cells isolated from these donors. Surprisingly, there was no effect of PMD on microglial cell yield, while cerebrospinal fluid pH had a positive correlation with the yield (Mizee et al., 2017). Next, a high RNA integrity number, reflecting no RNA degradation within the tissues, is an important factor to generate reliable and reproducible gene expression datasets. In brain tissue, RNA quality of controls has been reported to be significantly higher than in disease conditions, but overall RNA quality was independent of PMD up to 36 h (White et al., 2018). In addition, RNA quality is independent of storage time (White et al., 2018; Shah et al., 2019). RNA quality differences within one dataset might mask biologically relevant information. Computationally, it is possible to correct for RNA quality using a linear model framework (Gallego Romero et al., 2014). However, tissue selection based on RNA quality is an important step prior to downstream analysis. To summarize, RNA quality and microglia isolation procedures have larger effects on gene expression than the PMD. However, only a minimal amount of studies investigated the effect of PMD on gene expression; so far, these studies did not use high-throughput gene expression technologies.

## Proteomic Studies to Decode Cellular Heterogeneity

Proteomics is the study of the entire proteome in a cell, tissue, or organism. The proteome is dynamic and subject to posttranslational modifications, protein–protein interactions, synthesis, and degradation (Chandramouli and Qian, 2009; Aslam et al., 2017). Techniques to analyze specific proteins are Western blotting and enzyme-linked immunosorbent assay (ELISA); however, these allow the analysis of just a few proteins at a time (Aslam et al., 2017). Coons et al. (1941) were the first to use IHC in their study for the spatial detection of protein epitopes within a tissue section. Already in the 1990s, HLA-DR stainings were performed to study antigen representation in NAWM from MS patients compared to controls (Hayes et al., 1988). IHC is a technique that is still used; however, over the years, new methods have been developed for protein detection that allow a higher throughput or are combined with transcriptomics.

One protein detection technique with a higher throughput is mass cytometry. This technique is a combination of flow cytometry and mass spectrometry. Instruments to measure mass cytometry are cytometry by time-of-flight (CyTOF), CyTOF2, and the most recent one, Helios. Both sensitivity and throughput increased with each generation of instruments. Mass cytometry requires single-cell suspensions, which are incubated in a pool of antibodies conjugated to a unique, stable heavy-metal isotope (Figure 3). These isotopes are used as reporters, capable of measuring the gene expression of specific targets. Next, nebulized droplets are introduced into the inductively coupled argon plasma (ICP). Here, ions are liberated, and cells are atomized. Ions are filtered in the quadripole, which allows only the heavy-metal reporter ions to be distinguished by their variation in mass and to be quantified by CyTOF(2) or Helios. Up to 45 parameters can be detected, which is a large improvement compared to the 8–12 parameters that can be detected by flow cytometry (Bendall et al., 2012; Spitzer and Nolan, 2016). Mass cytometry does not only allow for the detection of proteins and their expression levels; also posttranslational modifications and proteolytic products can be determined. For example, the technique is able to measure phosphorylated (activated) states of proteins, which could provide insights in cellular behavior (Spitzer and Nolan, 2016). Mass cytometry thus contributes to the decoding of cellular heterogeneity. Ajami et al. (2018) used cell cytometry to characterize myeloid populations in EAE and other models for neurodegenerative diseases [Huntington’s disease (HD) and amyotrophic lateral sclerosis (ALS)]. Using CyTOF, Ajami et al. (2018) identified two CD11b+ myeloid populations in the healthy brain, while an extra CD11b+ myeloid population was detected in the CNS of EAE mice and in HD and ALS models. The first population (population A) was characterized by a CD317+ MHC-II–CD39lowCD86- profile; the second population (population B) was CD317+ MHC-II–CD39hiCD86+; and the third (population C) was CD317+MHC-II+CD39hiCD86+, indicating that populations B and C consisted of activated microglia. Furthermore, CD11c was only expressed by population C. During EAE progression, population C expanded, while in chronic EAE and in the recovery phase, a decrease in the number of cells in population C was observed. CyTOF revealed coexpression of granulocyte-macrophage colony-stimulating factor (GM-CSF) and TNFα in most of the cells in population C during EAE onset and peak of disease, while in HD and ALS models, the percentage of cells expressing these cytokines was low or absent. Another interesting observation was an increase in the expression of the signaling molecules pCREB and pMAPKAPK2 in populations B and C during the onset and subsequently during the peak of EAE. In addition, CyTOF revealed that CD49d (α4 integrin) and CD49a (α5 integrin) were only expressed on peripheral monocyte populations and not on CNS resident myeloid populations. Blocking CD49a expression attenuated EAE, highlighting the potential of CD49a as a therapeutic target (Ajami et al., 2018). This study confirms that mass cytometry is an interesting method to consider when studying cellular heterogeneity, as it is a single-cell method that can distinguish different populations based on (co)expression levels of transcription factors, signaling molecules, cytokines, and other proteins in a specific cell type.

Mass cytometry has a throughput of around thousand cells per seconds, which is much lower compared to flow cytometry, making the technique time consuming. Furthermore, as cells are ionized and atomized during mass cytometry and thus fully shattered, it is not possible to sort cells and collect them for further analysis (Bandura et al., 2009; Spitzer and Nolan, 2016).

New methods, such as cellular indexing of transcriptomes and epitopes by sequencing (CITE-seq) and RNA expression and protein sequencing assay (REAP-seq), have been developed that measure both cell-surface protein and gene expression levels in the same individual cell (Figure 3). Both techniques use DNA-barcoded antibodies to tag proteins of interest. These barcodes are unique oligonucleotides that contain an amplification primer and sequencing handle. After cell lysis, reverse transcription, and preamplifications steps, library preparation can be performed followed by single-cell sequencing (Peterson et al., 2017; Stoeckius et al., 2017). The difference between the two techniques is the way the antibody is conjugated to the DNA sequence. In CITE-seq, antibodies are conjugated to streptavidin, which is non-covalently bound to the oligonucleotide, while in REAP-seq, the antibody and oligonucleotide are conjugated by covalent bonds (Peterson et al., 2017; Todorovic, 2017). At the moment, both techniques are not able to detect intracellular proteins; however, due to the conjugation of antibody and barcode by covalent bonds, REAP-seq has potential for intracellular labeling of proteins in the future (Peterson et al., 2017). At present, up to 82 antibodies can be multiplexed at the same time, although it is likely that this number will increase as CITE-seq and REAP-seq are not obstructed by signal collision as is the case in flow and mass cytometry (Stoeckius et al., 2017).

Taken together, proteomic studies are of importance when investigating underlying mechanisms of diseases, as mRNA gene expression levels do not always reflect protein expression levels due to posttranslational modifications, protein trafficking, localizations, and protein–protein interactions. Mass cytometry could provide insights, as it is able to determine posttranslational modification and proteolysis products. On the other hand, since REAP-seq and CITE-seq can measure cell-surface protein and gene expression levels in parallel, they could also contribute to a better understanding of cell functioning and/or cellular heterogeneity.

## Discussion: Comparison of Transcriptomics, Spatial Detection, and Proteomic Methodologies. Which Method Should I Use?

Wide availability of (novel) technologies makes it challenging to choose the most appropriate method to apply to answer specific scientific questions. Here, we provide an overview of important properties per technology, which can facilitate the decision-making process (Table 1 and Supplementary Figure 1). All sequencing-based technologies are unbiased, facilitating novel discovery, since there is no need to preselect candidate genes.

**TABLE 1:** Comparison transcriptomics and proteomics technologies.

Target	Technology	Resolution (μM)	Sensitivity	Time needed to run method	Nr of targets detected simultaneously	Costs	Spatial context preserved	Requires candidate genes/proteins	Suitable for low abundant targets	Tissue type	Commercially available	Brief description of the chemistry	References methods
RNA	Microarray	–	++	+	++ (>100 genes)	+	N	Y	Y	F, FF	Low Input Quick Amp Labeling Kit (Agilent)	Barcoded microfluidic chip	Mycko et al., 2004; Hendrickx et al., 2017; Zheng et al., 2018
	BULKseq	–	+++	+++	+++	+	N	N	N	F, FF. FFPE	Lexogen QuantSeq 3’ mRNA-Seq kit, KAPA Stranded mRNA-Seq kit, NEB Next Ultra Directional RNA Library Prep Kit from Illumina	OligoDT primers or beads combined with barcode or UMI	Niu et al., 2019; Geirsdottir et al., 2019; Elkjaer et al., 2019; van der Poel et al., 2019; Rodríguez-Lorenzo et al., 2020
	LCMseq	+++	+++ (LCM-bulk seq) + (LCM-scRNAseq)	–	+++	+	Y	N	N	FF, FFPE, stained tissues	N	Infrared or ultraviolet guided laser capture microdis- section	Mycko et al., 2012; Shrestha et al., 2014; Datta et al., 2015; Guillot et al., 2015; Waller et al., 2016; Nichterwitz et al., 2016; Shrestha et al., 2017; Hendrickx et al., 2017; Foley et al., 2019
	scRNAseq - Full length cDNA	+++	–	++	+++	++	N	N	Y	F	N	OligoDT primer, template switching and tagmentation UMI and barcoded full length cDNA	Picelli et al., 2014; Mathys et al., 2017
	scRNAseq - 3′/5′ end cDNA	+++	–	+++	+++	++	N	N	Y	F	10X Genomics (chromium)	Microfluicic partitioning, UMI and barcoded 3/5 prime cDNA	Keren-Shaul et al., 2017; Geirsdottir et al., 2019; Hammond et al., 2019; Jordǎo et al., 2019; Masuda et al., 2019
	snRNAseq	+++	–	+++	+++	++	N	N	Y	F, FF	10X Genomics (chromium)	Microfluicic partitioning, UMI and barcoded 3/5 prime cDNA	Krishnaswami et al., 2016; Schirmer et al., 2019; Gerrits et al., 2020
	Spatial transcriptomics	–	–	++	+++	++	Y	N	N	FF	10X Genomics (Visium)	OligoDT probes barcoded on slide	Ståhl et al., 2016; Asp et al., 2017; Salmén et al., 2018; Gregory et al., 2020
	Slide-seq	+++	–	+	++	+	Y	N	N	FF	N	Barcoded microparticles on a rubber-coated glass coverslip	Eisenstein, 2019; Rodriques et al., 2019
	ISS	+++	Unknown	–	+	Unknown	Y	Y	N* *only possible if you extend the nr of probes per target	FF, FX	N	Padlock probes	Ke et al., 2013; Maïno et al., 2019
	ISS - Next generation ISS (CARTANA)	+++	–	–	++	+++	Y	Y	N* *only possible if you extend the nr of probes per target	FF, FFPE, FX	CARTANA	Padlock probes	CARTANA AB. 2020 URL: https://www.cartana.se/
	FISSEQ	+++	—	—	++	+	Y	N	N	FF, FFPE	ReadCoor Inc.	RNA is converted into cross-linked cDNA amplicons	Mitra et al., 2003; Lee et al., 2015
	MERFISH	+++	++	+	++	+	Y	Y	Y	FF	N (upcoming; Vizgen)	Probes	Chen et al., 2015; Xia et al., 2019; Wheeler et al., 2020
	smFISH	+++	+++	–	–	++	Y	Y	Y	FF, FFPE	PixelBioTech GmbH, Biosearch Technologies, Inc	Probes	Femino et al., 1998; Raj et al., 2008; Asp et al., 2017; Hammond et al., 2019; Schirmer et al., 2019
	RNAscope	+++	+++	+++	—	+	Y	Y	Y (RNA-scope 2.0)	FF, FFPE, FX	ACD Bio	Target-specific double Z probes	Wang et al., 2012 Advanced Cell Diagnostics, Inc. 2020. URL: https://acdbio.com/
Protein	Mass cytometry	+++	+++	+++	–	–	N	Y	N	F, FF, FFPE, FX	Novus Biologicals, Miltenyi Biotec, etc.	Antibodies conjugated to a heavy-metal isotope	Bendall et al., 2012; Spitzer and Nolan, 2016; Ajami et al., 2018
	IHC	+++	Depending on antibody/fixation method	++	—	–	Y	Y	N	F, FF, FFPE, FX	Y	Antibodies	Coons et al., 1941; van Horssen et al., 2012; Peferoen et al., 2015; Kuhlmann et al., 2017; Zrzavy et al., 2017; Van Wageningen et al., 2019
RNA and Protein	CITE-seq and Reap-seq	+++	+	++	Proteins; + Genes ++	+	N	Y candidate proteins	Y	F, FF, FX	Totalseq - Biolegend, Fluidigm	Cell-surface antibodies linked to oligonucleotide barcodes	Peterson et al., 2017; Baron et al., 2018; Stoeckius et al., 2017 Fluidigm 2020. URL: https://www.fluidigm.com/

*F, fresh; FF, fresh frozen; FFPE, formalin-fixed paraffin embedded; FX, fixed tissue samples. Technologies were compared on several parameters indicated by the following scoring range: −−−, −−, −, +, ++, +++, corresponding to very low, low, below average, average, high, very high, respectively. Other characteristics were indicated by Y, yes or N, no, referring to the presence or absence.*

High sensitivity is a major advantage of low-resolution transcriptomics. Increasing resolution and throughput generally decreases sensitivity (20%) due to a lower library complexity, which is a major drawback of high-resolution transcriptomics, and only allows the detection of variations in the most abundantly expressed genes. Sequencing methodologies differ in sequencing of whole transcripts (long reads) or sequencing of a small part of the transcript: the 3′ or 5′ end (short read). Short-read sequencing is not suitable to detect splicing variants and has a higher error rate, making annotating genes to the genome more complex compared to long-read sequencing. Therefore, long-read sequencing is more sensitive compared to short-read sequencing. However, in terms of examining heterogeneity and identifying microglia subtypes, high resolution is absolutely required. High-resolution transcriptomics is possible at the single-cell or single-nucleus level. snRNAseq has certain advantages above scRNAseq; first, nuclear RNA molecules can be obtained from frozen archived tissue samples, which can be very precisely classified for example into MS lesion types with matching HLA-DR scores. Furthermore, cell isolation and sorting is not required, which are the main factors causing cellular stress, which in turn can slightly change the transcriptome. Some cell subtypes are more vulnerable to cellular stress than others; therefore, nuclear RNA most likely represents a more reliable ratio of cell subtypes. Second, nuclear RNA molecules reflect active transcription, which might result in a more accurate view of the condition of interest. snRNAseq of microglia or other less abundant cell types benefits from enrichment strategies to obtain sufficient numbers for in-depth analysis. So far, studies have used a direct microglial enrichment strategy based on IRF8 sorting (van der Poel et al., 2019) or an indirect strategy by selecting a NEUN/OLIG2 negative population to select against major cell types of the brain: neurons and oligodendrocytes (Gerrits et al., 2020). Both single-cell and single-nuclei sequencing require extensive validation experiments to prove the existence and functionality of the detected subtypes, since spatial information is lacking.

**FIGURE 4:**
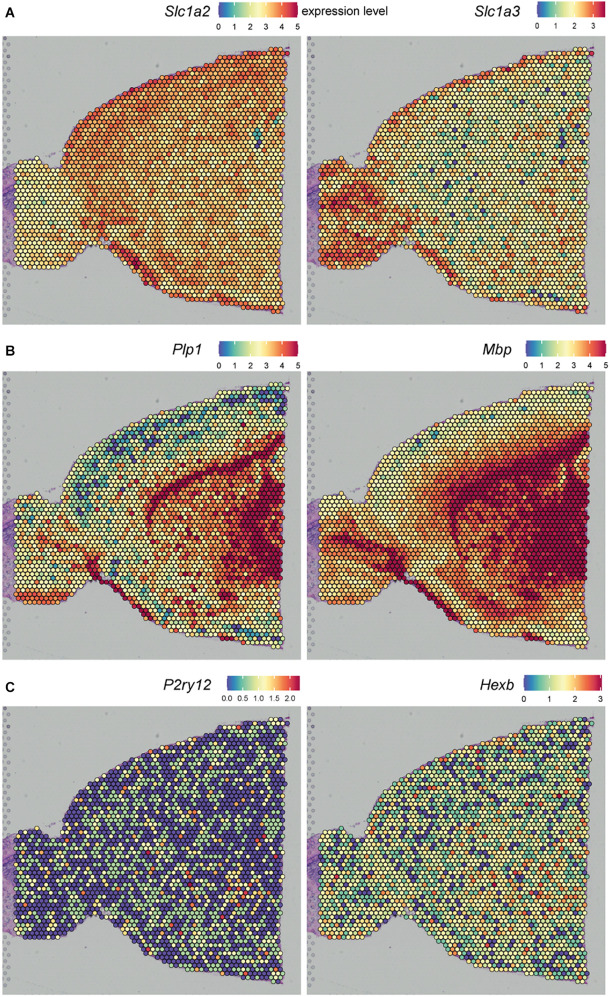
Spatial gene expression of **(A)** astrocyte, **(B)** oligodendrocytes, and **(C)** microglia-cell-specific marker genes, visualized in sagittal anterior control mouse brain. Dataset obtained from 10X Genomics.

Cell shape and localization often reflect functionality, which is already an old concept in biology. Therefore, spatial detection methods offer great possibilities to examine heterogeneity and functionality at the same time in a region specific manner, by using *ex situ* (spatial transcriptomics, slide-seq) or *in situ* (FISSEQ, MERFISH, ISS) technologies, reviewed here (Lee, 2017; Lein et al., 2017). However, can we obtain similar resolution and throughput as in single-cell/single-nuclei sequencing? Not at the moment. The resolution of *ex situ* spatial detection is 55 μM (1–10 cells/spot) for a commercially available method from 10X Genomics (Visium), as resolution is limited by printing distinguishable barcodes (Ståhl et al., 2016; Salmén et al., 2018; Gregory et al., 2020). That level of resolution could cause problems if your goal is to determine the contribution of the microglial transcriptome for a specific condition or disease, since microglia comprise only 10% of all brain cell types and since specific microglial subtypes are expected to represent an even smaller percentage. Compared to astrocyte and oligodendrocyte populations, these subsets likely provide too few RNA molecules to be detected and visualized in spatial transcriptomics in the mouse brain (Figure 4). Solving this issue would require higher resolution, higher sensitivity, and better computational deconvolution strategies. In comparison, slide-seq uses barcoded beads to increase the resolution to 10 μM (one to two cells/bead) and detection of genes is unbiased, although standardized description of the protocol is not available yet (Rodriques et al., 2019). Throughput of all spatial detection methodologies is limited by imaging speed; therefore, measuring the complete tissue section is often not possible or very expensive. *In situ* spatial detection methods are very suitable for regional validation of target genes in a cell-specific manner. ISS by CARTANA (Ke et al., 2013; Maïno et al., 2019) is a very promising and easy-to-use methodology, where individual genes are targeted by barcoded padlock probes, which is a more sensitive methodology compared to unbiased *in situ* approaches including FISSEQ (Lee et al., 2015). More specifically, the sensitivity of ST (7%), ISS (10%), and FISSEQ (0,005%) are all lower compared to single-cell or single-nuclei sequencing (20%). Other disadvantages of ISS are limited throughput and limited detected targets (up to 90 genes) restricted to predetermined target genes, and the design of novel padlock probes is costly. However, the next generation ISS allows detection of up to 600 genes and does not use padlock probes.

Proteomics technologies have some advantages above transcriptomic technologies. Proteomics is generally easier to apply because proteins are more stable than RNA molecules, although this might not be true for all posttranslational modifications. Most proteomics technologies are also cheaper compared to transcriptomics; however, exceptions are, for example, CyTOF, which requires the production of many antibodies. mRNA expression does not always predict protein expression levels in a cell. Therefore, proteomics could contribute to important insights in cell functioning. This will be a major advantage when the study goal is to find new effective drugs, since most therapeutics target proteins and not the mRNA, which makes proteomics crucial for drug development. For example, with a proteomic strategy, Ajami et al. (2018) discovered CD49a as a potential new drug target for MS. Furthermore, proteins are often adapted with posttranslational modifications such as phosphorylation, lipidation, and glycosylation, resulting in, for example, active forms of a protein with slightly changed folding or conformation. Proteomics technologies are able to distinguish the phosphorylated (active) state of a protein, from the non-phosphorylated (inactive) state (Spitzer and Nolan, 2016), which is highly important for biological data interpretation and cannot be detected with transcriptomics. High-throughput proteomic methodologies, such as REAP-seq and CITE-seq, can perform proteome and transcriptome analysis simultaneously, which allows to compare the levels of both molecules in one biological sample. Despite these major advantages, there are also disadvantages to this type of proteomics technique; often, not all proteins can be detected easily, and some technologies can only detect extracellular and not intracellular proteins. Moreover, protocols for proteomic methodologies are not commercially available, thus requiring more skills to robustly perform these technologies. Furthermore, proteomics can often detect a lower number of targets simultaneously compared to transcriptomics; however, this is expected to increase in the future.

## Future Perspectives

The recent advances in high-resolution gene and protein expression profiling of microglia in MS have contributed to the identification of MS signatures in microglia subtypes; it makes researchers aware of the heterogeneity present in MS tissues. Future studies should take this heterogeneity into account by analyzing the different lesion types and GM/WM independently. This selective approach is likely to result in more information about MS-associated microglia subtypes. Currently, specific markers to isolate these MS-associated microglia subtypes individually are lacking. The field would remarkably benefit from isolating these disease-associated subtypes and perform more functional assays in order to elucidate their exact role in MS. Moreover, creating a landscape of cell (sub)type specific datasets allows studying interactions between cell (sub)types, for example ligand receptor mapping (Cabello-Aguilar et al., 2020). Furthermore, it can help to identify the drivers behind spatial heterogeneity; is this solely determined by the environment or do cell intrinsic mechanisms also play a role? In the context of MS, this information can be linked with genome-wide association studies (GWAS), which provide information about single-nucleotide polymorphisms (SNPs) associated with higher MS risk (Patsopoulos et al., 2019).

Finally, to facilitate the extraction of biologically relevant information from these large datasets, public platforms should be generated to share datasets produced by different research groups, stratified by disease, region, and cell (sub)types. Three existing RNA sequencing databases are GOAD—now Brain-sat (Holtman et al., 2015a), Brain RNA-seq (Zhang et al., 2014), and Neuroexpresso (Mancarci et al., 2017), which can be used to obtain glial-cell-type specific information. Currently, region specific information is lacking in these databases. For analysis of distinct regions, the mouse (Erö et al., 2018) or human brain atlas (Sunkin et al., 2013) can be used to annotate brain regions in spatial transcriptomics datasets. The Allen Brain Atlas already extended their anatomic data with genomic data, combining microarray, ISH, and MRI datasets. Future databases should be extended with protein and spatial information. The next step would be to integrate such a database with information about chromatin states associated with transcription regulation, such as chromatin accessibility (ATAC-seq), specific histone marks (CHIP-seq), or interactions within the genome and chromatin conformation capture technologies (Hi-C, PLAC-seq). Recently, an MS study by Factor et al. (2020) showed that this type of analysis can result in valuable information, as this study showed that the enhancer elements regulating *BRD3* and *HEXIM1* are directly associated with an MS risk gene and that these enhancers were dysregulated in MS, contributing to remyelination failure. Region and cell (sub)type specific profiling, as reviewed here, can be applied to diseases where cellular heterogeneity is an important factor in disease progression. In addition, it would allow for the identification and targeting of (pathogenic) cellular subsets. These specific pathogenic subtypes can potentially be reprogrammed into a homeostatic or more beneficial state via targeted drugs or treatments. Thus, the use of these novel technologies will result in better insights in MS disease pathogenesis and potentially contribute to the identification of novel targets to treat MS.

## Author Contributions

AM and MW wrote the manuscript and designed the figures. BE and SK revised the manuscript. All the authors contributed to the article and approved the submitted version.

## Conflict of Interest

The authors declare that the research was conducted in the absence of any commercial or financial relationships that could be construed as a potential conflict of interest.

## Abbreviations

ALS: amyotrophic lateral sclerosis
ATAC-seq: assay for transposase-accessible chromatin sequencing
BBB: blood–brain barrier
CC: corpus callosum
CHIP-seq: chromatin immunoprecipitation sequencing
CITE-seq: cellular indexing of transcriptomes and epitopes by sequencing
CNS: central nervous system
CyTOF: cytometry by time-of-flight
EAE: experimental-autoimmune encephalomyelitis
ELISA: enzyme-linked immunosorbent assay
FACS: fluorescent-activated cell sorting
FF: fresh frozen
FPE: formalin-fixed paraffin-embedded
FISH: fluorescent *in situ* hybridization
FISSEQ: fluorescent *in situ* sequencing
GM: gray matter
GML: gray matter lesion
GO: Gene ontology
GWAS: genome-wide association study
HD: Huntington’s disease
HE: hematoxylin and eosin
ICP: inductively coupled argon plasma
IHC: immunohistochemistry
ISH: *in situ* hybridization
ISS: *in situ* sequencing
LCM: laser capture microdissection
LPC: lysolecithin
LPS: lipopolysaccharide
MERFISH: multiplexed error-robust fluorescent in situ hybridization
MRI: magnetic resonance imaging
MS: multiple sclerosis
NAGM: normal-appearing gray matter
NAWM: normal-appearing white matter
OPC: oligodendrocyte precursor cell
PMD: postmortem delay
qPCR: quantitative polymerase chain reaction
RCA: rolling circle amplification
REAPseq: RNA expression and protein sequencing assay
ROS: reactive oxygen species
RRMS: relapsing–remitting multiple sclerosis
scRNAseq: single-cell RNA sequencing
smFISH: single-molecule fluorescent *in situ* hybridization
SNP: single-nucleotide polymorphisms
snRNAseq: single-nucleus RNA sequencing
ST: spatial transcriptomics
TLR: toll-like receptor
UMI: unique molecular identifier
WM: white matter
WML: white matter lesion.
