# Supplementary material for: High-Resolution Transcriptomic and Proteomic Profiling of Heterogeneity of Brain-Derived Microglia in Multiple Sclerosis
Source: Front Mol Neurosci. 2020 Oct 22;13:583811. doi: 10.3389/fnmol.2020.583811 (PMC7654237; doi:10.3389/fnmol.2020.583811)
Supplement: Supplementary file 1 [file Table_1.DOCX]

**Supplementary figure 1:** This scheme represents the decisions that have to be considered when applying low compared to high resolution molecular technologies. The black boxes with questions can be answered by yes or no, indicated by a green and red arrow respectively, resulting in the most appropriate technology suitable to answer a specific research aim. These technologies include: single nuclei RNA sequencing (snRNAseq), single cell RNA sequencing (scRNAseq), whole tissue sequencing (BULKseq), Microarray, Mass cytometry, single molecule fluorescent in situ hybridisation (smFISH), RNA scope, immunohistochemistry (IHC), quantitative polymerase chain reaction (qPCR), laser capture microscopy (LCM-seq), spatial transcriptomics (ST), in situ sequencing (ISS), Multiplexed error-robust fluorescence in situ hybridization (MERFISH), Slide-seq, fluorescent in situ sequencing (FISSEQ), RNA expression and protein sequencing (REAP-seq) and ellular indexing of transcriptomes and epitopes by sequencing (CITE-seq).
