# Supplementary figures and images for: High-Resolution Transcriptomic and Proteomic Profiling of Heterogeneity of Brain-Derived Microglia in Multiple Sclerosis
Source: Front Mol Neurosci. 2020 Oct 22;13:583811. doi: 10.3389/fnmol.2020.583811 (PMC7654237; doi:10.3389/fnmol.2020.583811)

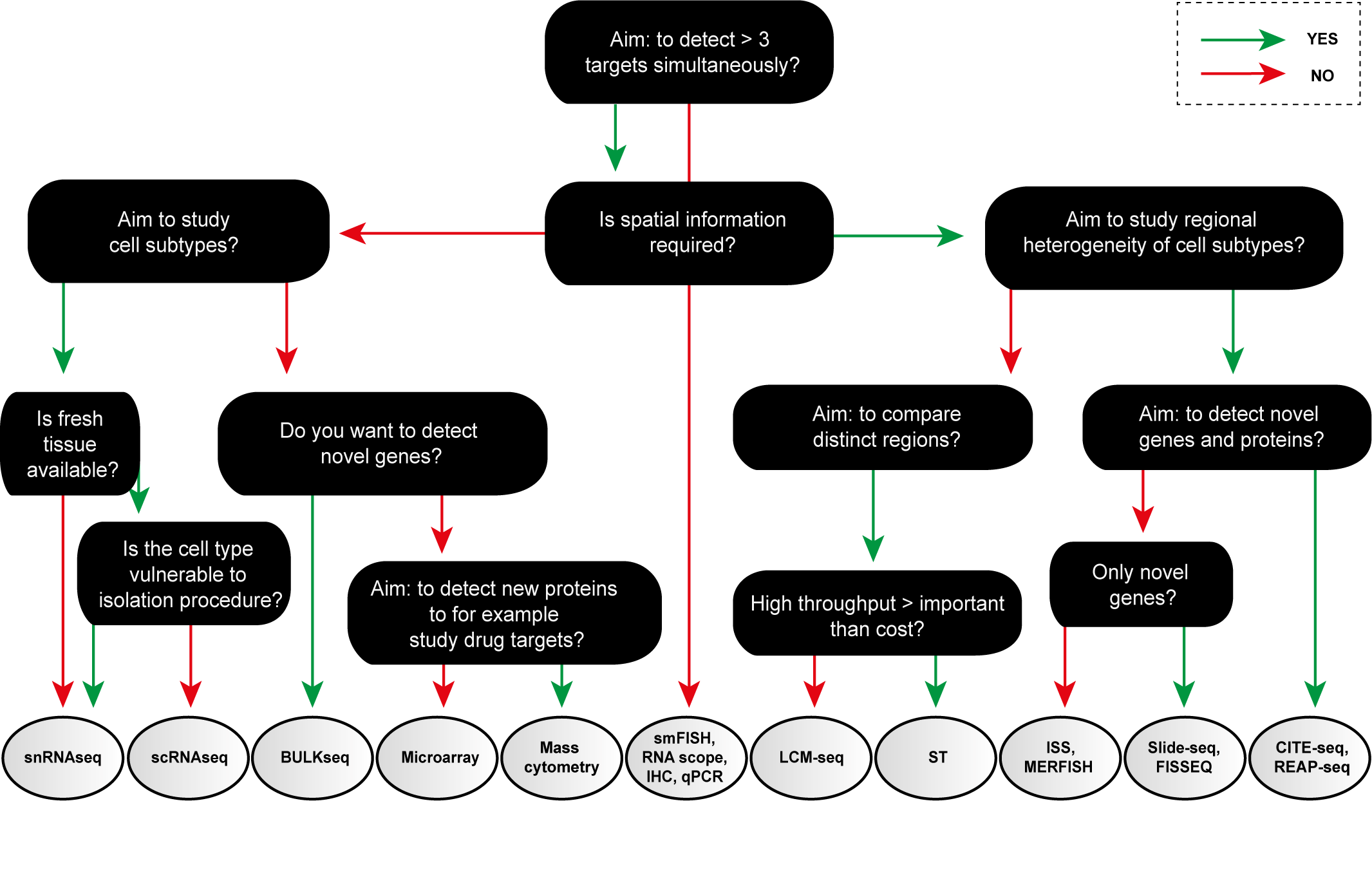

Supplement: Supplementary file 2 [file Image_1.TIF]
